# Synovial histopathology of psoriatic arthritis, both oligo- and polyarticular, resembles spondyloarthropathy more than it does rheumatoid arthritis

**DOI:** 10.1186/ar1698

**Published:** 2005-03-03

**Authors:** Elli Kruithof, Dominique Baeten, Leen De Rycke, Bernard Vandooren, Dirk Foell, Johannes Roth, Juan D Cañete, Annemieke M Boots, Eric M Veys, Filip De Keyser

**Affiliations:** 1 Department of Rheumatology, Ghent University Hospital, Ghent, Belgium; 2 Department of Pediatrics, University of Münster, Münster, Germany; 3 Institute of Experimental Dermatology, University of Münster, Münster, Germany; 4 Department of Rheumatology, Hospital Clinic de Barcelona, Universitat de Barcelona, Barcelona, Spain; 5 Department of Pharmacology, Organon NV, Oss, The Netherlands

## Abstract

At present only few biological data are available to indicate whether psoriatic arthritis (PsA) is part of the spondyloarthropathy (SpA) concept, whether it is a separate disease entity or a heterogeneous disease group with oligoarticular/axial forms belonging to SpA and polyarticular forms resembling rheumatoid arthritis (RA). To address this issue with regard to peripheral synovitis, we compared the synovial characteristics of PsA with those of ankylosing spondylitis (AS)/undifferentiated SpA (USpA) and RA, and compared the synovium of oligoarticular versus polyarticular PsA. Synovial biopsies were obtained from patients with RA, nonpsoriatic SpA (AS + USpA), and oligoarticular and polyarticular PsA. The histological analysis included examination(s) of the lining layer thickness, vascularity, cellular infiltration, lymphoid aggregates, plasma cells and neutrophils. Also, we performed immunohistochemical assessments of CD3, CD4, CD8, CD20, CD38, CD138, CD68, CD163, CD83, CD1a, CD146, α_V_β_3_, E-selectin, intercellular adhesion molecule-1, vascular cell adhesion molecule-1, S100A12, intracellular citrullinated proteins and major histocompatibility complex (MHC)–human cartilage (HC) gp39 peptide complexes. Comparing SpA (PsA + AS + USpA) with RA, vascularity, and neutrophil and CD163^+ ^macrophage counts were greater in SpA (*P *< 0.05), whereas lining layer thickness and the number of CD83^+ ^dendritic cells were greater in RA (*P *< 0.05). In RA, 44% of samples exhibited positive staining for intracellular citrullinated proteins and 46% for MHC–HC gp39 peptide complexes, whereas no staining for these markers was observed in SpA samples. We excluded influences of disease-modifying antirheumatic drug and/or corticosteroid treatment by conducting systematic analyses of treated and untreated subgroups. Focusing on PsA, no significant differences were observed between PsA and nonpsoriatic SpA. In contrast, vascularity (*P *< 0.001) and neutrophils were increased in PsA as compared with RA (*P *= 0.010), whereas staining for intracellular citrullinated proteins and MHC–HC gp39 peptide complexes was exclusively observed in RA (both *P *= 0.001), indicating that the same discriminating features are found in PsA and other SpA subtypes compared with RA. Exploring synovial histopathology between oligoarticular and polyarticular PsA, no significant differences were noted. Moreover, intracellular citrullinated proteins and MHC–HC gp39 peptide complexes, which are specific markers for RA, were observed in neither oligoarticular nor polyarticular PsA. Taken together, these data indicate that the synovial histopathology of PsA, either oligoarticular or polyarticular, resembles that of other SpA subtypes, whereas both groups can be differentiated from RA on the basis of these same synovial features, suggesting that peripheral synovitis in PsA belongs to the SpA concept.

## Introduction

Psoriatic arthritis (PsA) is a chronic inflammatory autoimmune disease that is characterized by inflammatory arthritis and skin psoriasis. It is commonly classified as a subtype of the spondyloarthropathy (SpA) concept, together with ankylosing spondylitis (AS), reactive arthritis, arthritis associated with inflammatory bowel disease, and undifferentiated spondyloarthropathy (USpA) [1]. Indeed, PsA shares a number of phenotypic characteristics with the other SpA subtypes, such as asymmetrical synovitis, enthesitis and familial aggregation. Typical SpA features such as sacroiliitis, presence of HLA-B27, uveitis and chronic inflammatory gut lesions are also found in PsA, albeit at a lower frequency than in other SpA subtypes [2-4]. Interestingly, the peripheral joint involvement is mostly asymmetrical and oligoarticular, as in SpA, but can also mimic rheumatoid arthritis (RA) with eventually destructive involvement of multiple small hand and feet joints [5]. Finally, skin and nail involvement, total ankylosis of destructed peripheral joints, predominant distal interphalyngeal joint involvement and arthritis mutilans sets PsA apart from both SpA and RA. Therefore, there is at present no clear consensus regarding whether PsA belongs to the SpA concept, whether it is a separate disease entity, or whether it is a heterogeneous disease group with oligoarticular and axial forms belonging to the SpA concept on the one hand and destructive, polyarticular forms resembling RA on the other [6,7]. This question will probably only be answered once the pathogenesis of the disease is fully elucidated, leading to an aetiological rather than phenotypical disease classification.

In previous histopathological synovial studies we considered PsA as part of the SpA concept and compared it as such with other inflammatory joint diseases. Although some early data are available on PsA synovial histology based on small studies of surgical or blind needle specimens, only few studies systematically evaluated PsA synovium obtained from actively inflamed joints [8,9]. Moreover, most of those studies used RA as a control and did not compare PsA and nonpsoriatic SpA, or oligoarticular and polyarticular PsA. In this context it must be highlighted that the few reports dealing with PsA synovium did not specify whether these patients had oligoarticular or polyarticular disease. The present study is the first to address these issues systematically and quantitatively in a large set of actively inflamed synovium samples by (1) conducting a detailed analysis of PsA synovitis in comparison with AS–USpA on the one hand and RA on the other, using a large panel of histopathological features; (2) comparing oligoarticular versus polyarticular PsA with respect to synovial features that discriminate between RA and SpA; and (3) analyzing the expression of S100A12, which was reported to be an interesting marker in PsA [10].

## Materials and methods

### Patients

In accordance with the aims of the study, three different patient cohorts were included. Cohort 1 was considered in the comparison of synovial histopathology between PsA, AS–USpA and RA. It included 22 patients with PsA having asymmetrical peripheral synovitis and skin psoriasis, and moreover fulfilling the European Spondyloarthropathy Study Group (ESSG) criteria [11], 28 nonpsoriatic SpA patients fulfilling the ESSG criteria (including 13 AS patients diagnosed according to the modified New York criteria [12] and 15 USpA patients), and 52 RA patients fulfilling the American College of Rheumatology (ACR) criteria [13]. Of the 22 PsA patients, six had a swollen joint count of more than 5 but with predominant involvement of the lower limbs and presence of other characteristics of SpA (i.e. enthesitis, sacroiliitis, familial history, etc.). None of them fulfilled the ACR criteria for RA [13]. Demographic and clinical features are summarized in Table 1.

Cohort 2 included a large number of PsA patients (*n *= 45), permitting us to compare synovial histopathology between oligoarticular and polyarticular disease. Of these patients 28 had oligoarticular disease with involvement of fewer than 5 peripheral joints, and the remaining 17 patients had polyarticular disease (≥ 5 joints). Demographic and clinical features are summarized in Table 2.

Cohort 3 was considered in the analysis of S100A12 by immunohistochemistry of synovial tissue. It included eight PsA patients (fulfilling the ESSG criteria [11]), 12 nonpsoriatic SpA patients (according to the ESSG criteria) and 20 RA patients fulfilling the ACR criteria [13]. The demographic and clinical features of these patients are summarized in Table 3.

Since we demonstrated previously that synovial histopathology is influenced by local disease activity in SpA and RA, all included patients were required to have a clinical effusion of at least one knee joint to perform needle arthroscopy with synovial biopsy sampling [14]. Written informed consent was obtained from all patients before their inclusion in the study, which was approved by the Ethics Committee of the Ghent University Hospital.

### Synovial histopathology

Synovial biopsies (16 per patient) were obtained by needle arthroscopy of the knee as described previously [15]. Eight biopsies were stored in formaldehyde and embedded in paraffin, and eight biopsies were snap frozen and mounted in Jung tissue freezing medium (Leica Instruments, Nussloch, Germany) and utilized for immunohistochemistry. The procedure for histological and immunohistochemical analysis of the different markers was extensively described and validated previously [14,16-20]. Briefly, paraffin-embedded biopsies were stained with haematoxylin and eosin for histological analysis, including mean synovial lining layer thickness (score: 1 = mean of 1–2 cell layers, 2 = mean of 3–5 cell layers, 3 = mean of >5 cell layers), vascularity of the sublining layer, global cellular infiltration of the sublining layer, and presence of lymphoid aggregates, plasma cells and polymorphonuclear cells (PMCs). Frozen sections of the synovial biopsies were stained with the following antibodies (from Dako [Glostrup, Denmark], unless otherwise stated): anti-CD3 (T cells, clone UCHT1), anti-CD4 (T-helper cells, clone MT310), anti-CD8 (cytotoxic T cells, clone DK25), anti-CD20 (B cells, clone L26), anti-CD38 (plasma cells, clone AT13/5), anti-CD138 (plasma cells, clone CBL455; Chemicon, Temecula, CA, USA), anti-CD68 (pan-macrophage marker expressed on monocytes and macrophages, clone EBM11), anti-CD163 (scavenger receptor expressed on resident tissue macrophages, clone Ber-MAC3), anti-CD83 (dendritic cells, clone HB15A; Immunotech SA, Marseille, France), anti-CD1a (interdigitating dendritic cells, clone NA1/34), anti-CD146 (endothelial cells, clone P1H12; Chemicon), anti-α_V_β_3 _(CD51/CD61, integrin expressed on endothelial cells, fibroblasts, osteoclasts, etc., clone LM609; Chemicon), anti-E-selectin (CD62E, endothelial leucocyte adhesion molecule 1, clone 1.2B6), anti-ICAM-1 (CD54, intercellular adhesion molecule [ICAM]-1, clone 6.5B5), anti-VCAM-1 (CD106, vascular cell adhesion molecule [VCAM]-1, clone 1.4C3), the rabbit polyclonal anti-L-citrulline antibody (citrullinated peptides; Biogenesis, Poole, UK), and Mab-12A (detecting major histocompatibility complex [MHC] class II–human cartilage [HC] gp39 peptide complexes; NV Organon, Oss, The Netherlands) [18,21]. Immunostaining for S100A12 (calgranulin C) was performed with polyclonal affinity-purified rabbit antisera against human S100A12 (a S100A12) [22].

It should be noted that the scavenger receptor CD163 identifies a subset of CD68+ macrophages that is specifically increased in SpA synovium and gut, and which could play an important role in innate immune inflammation of these tissues [16,19].

After incubation with the primary antibody, sections were sequentially incubated with a biotinylated second antibody, a streptavidine–horseradish peroxidase link, and finally with amino-ethyl-carbazole substrate as chromogen. Parallel sections were incubated with irrelevant isotype and concentration matched monoclonal antibody as negative control. Sections were coded and analyzed semiquantitatively on a 4-point scale (0–3) by two independent observers who were blinded to diagnosis and clinical data. Because the numbers of positive cells per synovial section for citrullinated peptides and Mab-12A were too small to be scored semiquantitatively, these parameters were scored as present or absent. The analysis included all areas of the eight biopsies and a global score was given for each parameter, using a semiquantitative 4-point scale (0 = lowest level of expression, 3 = highest level of expression) [14,16-21]. Because some histological markers are more abundant than others, the scoring system was calibrated for each marker separately by examining a representative number of samples. The scores obtained by the two observers were concordant in more than 95% of cases. In the event of discordant scores, which differed by a maximum of 1 point, the mean of the two scores was used.

### Statistics

Because the semiquantitative data are nonparametric, these data are presented as median (range). Differences between groups were analyzed using the nonparametric Mann–Whitney U-test. For markers scored as present or absent, the χ^2 ^test was used. Bonferroni adjustment of alpha was performed where indicated. *P *< 0.05, after Bonferroni correction, was considered statistically significant.

## Results

### Synovial histopathology of spondyloarthropathy versus rheumatoid arthritis

We previously established the differences between SpA (including PsA fulfilling ESSG criteria) and RA in terms of global synovial histology and infiltrating cell populations [14,16]. Therefore, in patient cohort 1 we compared the following synovial features between the pooled SpA group (50 samples) and the RA group (52 patients): lining layer thickness, vascularity, global cellular infiltration, lymphoid follicles, plasma cells, PMCs, CD3, CD4, CD8, CD20, CD38, CD138, CD68, CD163, CD1a, CD83, CD146, α_V_β_3_, E-selectin, ICAM-1, VCAM-1, intracellular citrullinated proteins and MHC–HC gp39 peptide complexes.

As shown in Table 4, the lining layer thickness (*P *= 0.006) and number of CD83+ dendritic cells (*P *= 0.006) were significantly greater in RA than in SpA. There was also a trend toward greater CD38+ plasma cell infiltration in RA than in SpA (*P *= 0.060). On the contrary, vascularity (*P *< 0.001), infiltration with PMCs (*P *= 0.008), and the presence of CD163+ macrophages in the lining layer (*P *= 0.033) and sublining layer (*P *= 0.031) were higher in SpA (although the medians for PMC infiltration did not differ, reflecting that only a subset of SpA samples was characterized by high degree of PMC infiltration). In agreement with previous studies [16,19], the increase in the CD163+ macrophage subset in SpA was not parallalled by changes in the global number of macrophages, as detected by the panmacrophage marker CD68. ICAM-1 staining in the synovial lining layer (*P *= 0.025), but not in the sublining or on the vascular endothelium, was also higher in SpA than in RA. Intracellular citrullinated proteins and MHC–HC gp39 peptide complexes were observed only in RA patients (44% positive for citrullinated proteins and 46% positive for MHC–HC gp39 peptide complexes), with absence of staining for these markers in the SpA samples (for both: *P *< 0.001).

**Figure 1:**
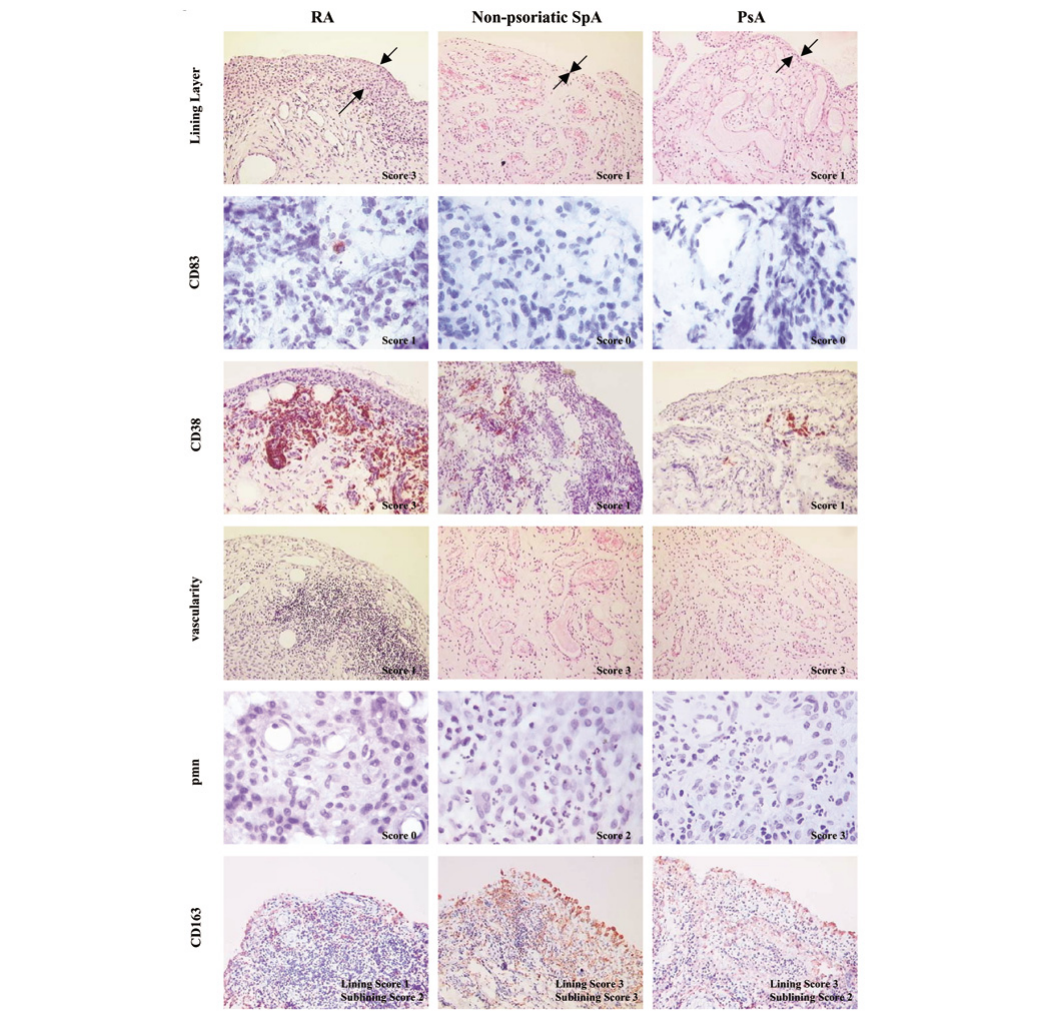
Synovial histology in patients with rheumatoid arthritis (RA), psoriatic arthritis (PsA) and nonpsoriatic spondyloarthropathy (ankylosing spondylitis [AS] + undifferentiated spondyloarthropathy [USpA]). Synovial biopsies from RA, PsA and spondyloarthropathy (SpA; AS/USpA) patients were scored on a semiquantitative scale (0–3) by two independent observers. Representative sections of RA and PsA and nonpsoriatic SpA synovium are shown, and the corresponding semiquantitative score for each picture is indicated. The evaluated parameters included synovial lining layer thickness, CD83^+ ^dendritic cells, CD38^+ ^plasma cells, degree of vascularity, number of neutrophils (polymorphnuclear neutrophils [pmn]) and CD163^+ ^macrophages.

There were no other significant differences between both groups. Taken together these data confirm our previous observations and indicate that the samples selected for the study are representative of the global histopathological picture of SpA and RA. The differences are illustrated in Fig. 1.

### Influence of disease-modifying antirheumatic drug and steroid treatment on synovial histopathology

Because the studied disease groups in cohort 1 received different therapies (Table 1) and because previous longitudinal studies with paired biopsy sampling have demonstrated the effect of disease-modifying antirheumatic drugs (DMARDs) and systemic corticosteroids on synovial histopathology [23-27], we assessed whether DMARD and/or systemic corticosteroid therapy could bias a cross-sectional histopathological evaluation of clinically involved joints.

As shown in more detail in Table 1, six out of 22 PsA patients, eight out of 28 AS–USpA patients, and 25 out of 52 RA patients were treated with DMARDs and/or systemic corticosteroids. For the RA group, we compared patients with versus those without DMARD treatment, as well as patients with versus those without corticosteroid treatment with respect to the synovial features mentioned above. Only endothelial VCAM-1 (higher in RA with than in RA without DMARD treatment: 2.0 [0–3.0] versus 0 [0–3.0]; *P *= 0.035) and endothelial E-selectin (lower in RA with than in RA without corticosteroid treatment: 0.5 [0–1.5] versus 1.5 [0–3.0]; *P *= 0.017) were significantly different as a function of treatment.

For the SpA group (PsA + AS + USpA), we compared 13 DMARD-treated patients with 37 patients who received no DMARD treatment. Only ICAM-1 expression was significantly different as a function of DMARD treatment (lower in SpA with than in SpA without DMARD treatment: 2.5 [1.0–3.0] versus 3.0 [1.5–3.0] for the lining layer [*P *= 0.033], and 1.0 [0–2.5] versus 2.5 [0–3.0] for the sublining layer [*P *= 0.003]).

Focusing on the PsA group, a comparison of patients with versus those without DMARD treatment for the same parameters revealed no significant differences.

### Synovial histopathology of psoriatic arthritis versus rheumatoiad asthritis and versus ankylosing spondylitis–undifferentiated spondyloarthropathy

Having confirmed histopathological differences between RA and SpA synovitis and having excluded a systemic bias introduced by therapy, we then focused more specifically on PsA. Using the same histological markers, in patient cohort 1 we compared 22 PsA with 52 RA synovia samples. The degree of synovial vascularity was higher in PsA (median 2.5 [range 1.0–3.0]) than in RA (1.5 [1.0–3.0]; *P *< 0.001). Similarly, the presence of PMCs was more pronounced in PsA (0.5 [0–3.0]) than in RA (0 [0–2.5]; *P *= 0.010). Intracellular citrullinated proteins and MHC–HC gp39 peptide complexes were not observed in PsA, whereas 44% of RA samples were positive for citrullinated proteins and 46% were positive for MHC–HC gp39 peptide complexes (for both: *P *= 0.001). There was no significant difference for the other investigated markers, including E-selectin and CD68, which were previously reported to be different between both diseases [8]. Performing a similar analysis in function of AS–USpA, we compared the 22 PsA samples with 28 nonpsoriatic SpA samples (13 AS and 15 USpA); none of the investigated markers was significantly different between both groups.

### Oligoarticular versus polyarticular psoriatic arthritis

It was previously suggested that PsA could comprise different subsets, including an oligoarticular subtype that resembles SpA and a polyarticular form mimicking RA. To address this issue, we constituted a new PsA cohort (patient cohort 2) including 28 patients with oligoarticular disease (<5 swollen joints) and 17 patients with polyarticular PsA (≥ 5 swollen joints). We compared the PsA subgroups with respect to those synovial features that appeared to discriminate best between SpA and RA in the previous experiments or that have been reported in the literature [8]: lining layer hyperplasia, vascularity, PMCs, CD163+ and CD68+ macrophages, and expression of E-selectin. As shown in Table 5, no significant differences in these parameters were observed between oligoarticular and polyarticular PsA. Again, intracellular citrullinated proteins and MHC–HC gp39 peptide complexes – two markers specific for RA – were not observed in either the oligoarticular or polyarticular PsA samples.

### S100A12

**Figure 2:**
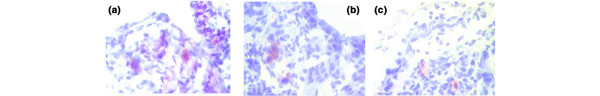
Synovial expression of S100A12 in psoriatic arthritis (PsA), nonpsoriatic spondyloarthropathy (SpA; ankylosing spondylitis [AS] + undifferentiated spondyloarthropathy [USpA]) and rheumatoid arthritis (RA). **(a) **PsA: lining = 0, sublining = 1 (original magnification 640×); **(b) **nonpsoriatic SpA: lining = 0, sublining = 2 (original magnification 640×); and **(c) **RA: lining = 0, sublining = 2 (original magnification 640×).

Finally, we investigated whether, apart from the well known histopathological markers evaluated in the previous experiments, additional specific immunohistochemical stainings were helpful in the evaluation of PsA synovitis compared with nonpsoriatic SpA and RA. Based on the greater infiltration with PMCs in SpA than in RA, and a recent report on the granulocyte calcium-binding protein S100A12 in PsA [10], we assessed the expression of S100A12 in synovium of patients with PsA (*n *= 8), nonpsoriatic SpA (*n *= 12) and RA (*n *= 20; patient cohort 3; Table 3). Cellular staining for S100A12 was observed in the sublining layer in all three patient subgroups, whereas staining in the lining layer was only occasionally observed (Fig. 2). Confirming the findings of the previous study [10], S100A12 expressing cells were found essentially in perivascular infiltrates, either adjacent to small blood vessels or adhering to endothelial cells. However, this staining pattern was observed in all groups without clear distinction between PsA, nonpsoriatic SpA and RA. This difference may be accounted for by the fact that, in the study conducted by Foell and coworkers [10], the disease duration of the patients at study entry was shorter, and none of the PsA-patients received any DMARDs or steroids. Moreover, there was no significant difference in the degree of staining between PsA (median 1 [range 0–3]) and nonpsoriatic SpA (1, [0–2]; *P *= 1.000) or RA (median 0 [0–3]; *P *= 0.430). Pooling of the PsA and nonpsoriatic SpA samples in one SpA group did not result a difference between SpA and RA either (*P *= 0.096).

## Discussion

Because the synovial membrane is one of the primary tissue targets in PsA, detailed histopathological analysis of PsA synovitis might be a useful approach to gain new insights into this disease and to analyze biological similarities with or differences from other chronic inflammatory joint disorders such as RA and SpA. This is of particular importance because PsA is thought to resemble either RA or SpA, depending on the clinical pattern of peripheral joint involvement, with respectively polyarticular symmetrical disease and oligoarticular asymmetrical involvement. In this context, a detailed analysis of the synovial histopathology in PsA not only may provide new insights in the disease process but also may help to provide a biological rationale for the classification of PsA. However, until now such analyses are lacking.

Synovial histopathology in PsA is generally characterized by neovascularization, and inflammatory infiltration with predominantly mononuclear cells (T lymphocytes, B lymphocytes and plasma cells, and macrophages), although PMCs can also be detected [28]. Mild to moderate synovial lining hyperplasia is observed in a considerable percentage of cases. Whereas these characteristics are found as well in other types of inflammatory arthritis, the availability of synovial biopsies from needle arthroscopy and new histopathological tools (monoclonal antibodies, RNA probes) have permitted a more detailed comparison of PsA and RA synovitis. One of the most prominent differences was the higher degree and typical morphology of synovial vascularity, which appeared to be related to angiogenic factors such as vascular endothelial growth factor, matrix metalloproteinase (MMP)-9 and angiopoietins, and was accompanied by a higher expression of E-selectin [8,29,30]. Lining layer hyperplasia appeared to be more pronounced in RA, both by altered apoptosis of lining cells as by increased presence of CD68+ macrophage-like synoviocytes, although macrophage-derived pro-inflammatory cytokines such as tumour necrosis factor-α were also found abundantly in PsA synovium [8,31-33]. Apart from a difference in CD68+ macrophages and the somewhat unexpected presence of PMCs in PsA synovitis [8,28], no major differences in infiltrating cell populations were described between PsA and RA. Although these differences between PsA and RA are of interest, it should be highlighted that these reports did not clearly distinguish between different clinical subtypes of PsA and did not compare PsA with SpA.

Considering that the oligoarticular form of PsA might belong to the SpA concept, both the present study and previous reports of our group indicate similar differences between SpA and RA as those described between PsA and RA: higher lining layer thickness in RA, but more pronounced vascularity, presence of polymorphonuclear cells and presence of the CD163+ macrophage subset in SpA.[14,16,19]. However, we could not confirm earlier reported differences in the presence of CD68+ macrophages between the disease groups [8]. These findings of the present study are in agreement with our previous reports in SpA [16,19], in which we indicated that the increased synovial infiltration with CD163+ macrophages – a subset that may play an important role in innate immune inflammation – is not paralleled by an increase in global macrophage number, as detected using the pan-macrophage marker CD68. This is also in agreement with a recent independent study [34] comparing PsA with RA, in which no difference was found in the number of CD68+ macrophages between the diseases.

In order to confirm whether similarities between SpA and PsA exist, as suggested by these independent studies, the present study provides a direct comparison of oligoarticular PsA with RA and with nonpsoriatic SpA. Our findings clearly demonstrate the resemblance between oligoarticular PsA and SpA synovium, whereas oligoarticular PsA synovium is significantly different from RA in terms of lining layer hyperplasia and PMC infiltration. Having previously indicated the importance of local disease activity but not disease duration in the assessment of synovial histopathology, the present study furthermore provides evidence that the obtained results are not biased by differences in treatment between the groups. Confirming the observations of other recent studies [19,35,36], these data certainly do not suggest that DMARD and/or corticosteroid treatment have no influence on synovial histopathology [23-27] but they indicate that global synovial histopathology is not altered in the case of persistent synovitis despite DMARD and/or systemic corticosteroid treatment.

Bearing in mind that subclassification of PsA is based on the pattern of peripheral joint involvement [6,7], it is surprising that, until now, no synovial histology data were available detailing oligoarticular PsA, which is thought to be related to SpA, and polyarticular PsA, which can mimic RA. Using histological parameters that discriminate between SpA and RA, such as lining layer thickness, degree of vascularity, presence of PMCs and CD163 expression, we were unable to demonstrate any significant differences between oligoarticular and polyarticular PsA. In addition, earlier reported markers distinguishing PsA from RA, such as CD68 and E-selectin, appeared not to be differentially expressed between oligoarticular and polyarticular PsA [8,37]. Considering the discrepancies with regard to published data on CD68 and E-selectin expression between PsA and RA, one should consider potential biases in study populations or in treatment schedules. Moreover, intracellular citrullinated proteins and Mab-12A staining, which are highly specific for RA and were found in approximately half of the RA synovia [17,18], were not detected in the PsA cohorts. These data provide the first clear evidence that polyarticular PsA does not exhibit RA-specific synovial features and that the synovial histopathology of PsA, either oligoarticular or polyarticular, is more closely related to SpA than to RA.

Apart from global histological features, a number of studies have analyzed more specific molecular systems in PsA synovium, including S100 proteins. The calcium-binding granulocyte protein S100A12 was recently described in PsA synovium [10]: although the number of analyzed samples was too small to allow statistical comparison, a specific expression in PsA as compared with RA was suggested. In the present study we confirmed both the presence and the staining pattern of S100A12 in PsA synovium, but neither synovial tissue analysis nor synovial fluid measurements identified differences between PsA, SpA and RA. Two other S100 proteins (S100A8 and S100A9, respectively named myeloid-related protein [MRP]8 and MRP14), which are found in both infiltrating PMCs and monocytes, were recently described in different types of inflammatory arthritis [36-40]. Similar to the findings of the present study, the expressions of these MRPs in synovium and synovial fluid were different between SpA and RA but not between PsA and nonpsoriatic SpA [36]. Furthermore, we recently provided evidence that the effect of tumour necrosis factor-α blockade with infliximab on MRPs, as well as on global histological features, was similar in PsA and in nonpsoriatic SpA, further supporting the concept that peripheral joint disease is closely related in both diseases [41,42].

Nevertheless, the data provided by the present study and the resulting hypothesis require some critical comments. First, it should be noted that all patients included in the present study had an active knee synovitis, which could bias the study population by excluding polyarticular PsA with predominant or exclusive involvement of hand and feet joints. In addition, DMARD therapy has been shown to represent a confounding factor in terms of the pattern and number of swollen joints in established PsA; patients with polyarticular joint involvement may therefore be under-represented in our patient cohort [7]. Moreover, because occasional evolution to polyarticular disease suggests that PsA is a more systemic disease than is nonpsoriatic SpA, it should be further investigated whether there are specific histological alterations in clinically uninvolved joints in PsA versus AS and USpA. Second, more sensitive scoring (digital image analysis versus semiquantitative scoring) or detection methods might have revealed small differences that could have been overlooked in the present study. However, such small differences should be considered in the light of their likelihood of being reproduced on the one hand and their biological relevance on the other [43].

A third criticism of the present study is that it addressed a wide range of histopathological markers that are known to discriminate either PsA or SpA from RA, but the resulting concept requires confirmation by studies of cellular and molecular players, which are crucially involved in the pathogenesis of peripheral synovitis. Therefore, novel mediators identified *in vitro *or in animal models of PsA should be further evaluated. Considering the specific radiological features of peripheral joint involvement in PsA, an interesting issue – which was not addressed in the present study – is the synovial mechanism involved in cartilage and/or bone destruction. However, we recently investigated a set of metalloproteinases and their inhibitors in a similar study [35] that indicated that there was no difference in their synovial expression between SpA and RA. Surprisingly, the minor differences between PsA and nonpsoriatic SpA indicated less pronounced expression of MMP-1 and MMP-2 in PsA.

Another recent study [44] demonstrated that synovial osteoclasts and the receptor activator of nuclear factor-κB (RANK)/RANK ligand system, which are known to play a crucial role in bone degradation in RA, were also clearly present in PsA synovium. However, a systematic comparison of synovial osteoclasts and RANK/RANK ligand in PsA, SpA and RA remains to be conducted. Taken together, these three issues indicate that the absence of histopathological differences between PsA and non-psoriatic SpA in the present study does not indicate that these conditions are similar. Further studies are needed to address these issues in greater detail.

## Conclusion

The present study is the first to provide detailed comparisons of PsA synovitis with both RA and SpA, and of oligoarticular with polyarticular PsA synovitis. It indicates that the synovial histopathology of PsA, either oligoarticular or polyarticular, resembles SpA more than it does RA. These biological data do not support the clinical subclassification of PsA in a polyarticular RA-like and an oligoarticular SpA-like subtypes, and more importantly, they emphasize the need of more specific analyses of cellular and molecular characteristics, especially those that are involved in cartilage and bone pathology, to unravel the pathogenetic and phenotypic differences and similarities.

## Abbreviations

ACR = American College of Rheumatology; AS = ankylosing spondylitis; DMARD = disease-modifying antirheumatic drug; ESSG = European Spondyloarthropathy Study Group; HC = human cartilage; ICAM = intercellular adhesion molecule; MHC = major histocompatibility complex; MMP = matrix metalloproteinase; MRP = myeloid-related protein; PMC = polymorphonuclear cell; PsA = psoriatic arthritis; RA = rheumatoid arthritis; RANK = receptor activator of nuclear factor-κB; SpA = spondyloarthropathy; USpA = undifferentiated spondyloarthropathy; VCAM = vascular cell adhesion molecule.

## Competing interests

The author(s) declare that they have no competing interests.

## Authors' contributions

EK collected the samples, participated in the immunohistochemistry, performed statistical analysis and the interpretation of the study, and prepared the manuscript. DB designed the study, participated in its coordination, participated in the interpretation of the results, and drafted the manuscript. LDR participated in the collection of the samples, in the immunohistochemistry and in the interpretation of the results. BV participated in the collection of the samples and in the interpretation of the results. DF provided the polyclonal affinity-purified rabbit antisera against human S100A12 and participated in the interpretation of the results. JR provided the polyclonal affinity-purified rabbit antisera against human S100A12 and participated in the interpretation of the results. JC participated in the collection of the samples and in the interpretation of the results. AB provided the antibody detecting the MHC class II-HC gp39 peptide complexes and participated in the interpretation of the results. EMV supervised the collection of the samples as well as the design of the study. FDK participated in the design of the study and in its coordination, and participated in the interpretation of the results. All authors read and approved the final manuscript.

## Figures and Tables

**Table 1:** Demographic and clinical features of patients with PsA, nonpsoriatic SpA (AS + USpA) and RA.

Parameter	PsA	Nonpsoriatic SpA	RA
Number of patient	22	28	52
Sex (male/female)	14/8	22/6	25/27
Age (years)	45.6 ± 11.0	43.3 ± 13.7	54.0 ± 14.9
Disease duration (years)	10.1 ± 11.5	8.4 ± 9.8	4.9 ± 6.2
Number of swollen joints	4.1 ± 4.0	3.7 ± 4.0	10.5 ± 6.8
Serum CRP (mg/dl)	2.1 ± 1.9	4.0 ± 5.8	6.0 ± 7.4
ESR (mm/hour)	23.6 ± 22.2	30.1 ± 30.5	35.7 ± 27.1
Rheumatoid factor (+/-)	0/21	1/26	36/13
NSAID (+/-)	15/7	25/3	38/14
DMARD (+/-)	6/16	7/21	21/31
Corticosteroids (+/-)	1/21	2/26	12/40

The nonpsoriatic SpA subgroup includes 13 patients with AS and 15 with USpA. Values are expressed as mean ± standard deviation or as numbers. AS, ankylosing spondylitis; CRP, C-reactive protein; DMARD, disease-modifying antirheumatic drug; ESR, erythrocyte sedimentation rate; NSAID, nonsteroidal anti-inflammatory drug; PsA, psoriatic arthritis; RA, rheumatoid arthritis; SpA, spondyloarthropathy; USpA, undifferentiated spondyloarthropathy.

**Table 2:** Demographic and clinical features of patients with oligoarticular and polyarticular PsA.

Parameter	Oligoarticular PsA	Polyarticular PsA
Number of patients	28	17
Sex (male/female)	20/8	9/8
Age (years)	42.6 ± 10.9	43.7 ± 10.7
Disease duration (years)	7.4 ± 8.4	11.9 ± 11.1
Number of swollen joints	1.7 ± 1.0	9.2 ± 4.2
Serum CRP (mg/dl)	2.3 ± 3.6	3.4 ± 2.2
ESR (mm/hour)	19.2 ± 18.2	40.8 ± 21.1
Rheumatoid factor (positive/negative)	0/28	0/17
Sacroiliitis (≥grade II; +/-)	2/15	4/12
Enthesitis (+/-)	6/14	10/6
Hand joint involvement (+/-)	9/12	14/3
Symmetrical joint involvement (+/-)	4/19	10/7
DIP joint involvement (+/-)	8/11	10/7
NSAID (+/-)	21/6	15/1
DMARD (+/-)	8/19	10/6
Corticosteroids (treated/untreated)	2/25	3/13

Demographic and clinical features of patients with oligoarticular and polyarticular PsA. Values are expressed as mean ± standard deviation or as numbers. CRP, C-reactive protein; DIP, distal interphalangeal joint; DMARD, disease-modifying antirheumatic drugs; ESR, erythrocyte sedimentation rate; NSAID, nonsteroidal anti-inflammatory drug.

**Table 3:** Demographic and clinical features of patients with PsA, nonpsoriatic SpA and RA

Parameter	PsA	Nonpsoriatic SpA	RA
Number of patients	8	12	20
Sex (male/female)	4/4	9/3	8/12
Age (years)	45.1 ± 7.1	39.7 ± 8.9	58.9 ± 17.2
Disease duration (years)	6.0 ± 9.7	6.6 ± 6.0	4.5 ± 7.9
Number of swollen joints	3.0 ± 1.9	2.6 ± 3.1	11.7 ± 8.3
Serum CRP (mg/dl)	6.2 ± 6.5	4.3 ± 5.1	7.3 ± 7.4
ESR (mm/hour)	36.4 ± 34.7	23.8 ± 25.2	44.2 ± 28.9
Rheumatoid factor (+/-)	0/8	0/11	14/6
DMARD (+/-)	3/3	8/4	8/12
Corticosteroids (+/-)	1/5	0/12	9/11

The nonpsoriatic SpA subgroup includes six AS and six USpA patients. Values are expressed as mean ± standard deviation. AS, ankylosing spondylitis; CRP, C-reactive protein; DMARD, disease-modifying antirheumatic drug; ESR, erythrocyte sedimentation rate; NSAID, nonsteroidal anti-inflammatory drug; PsA, psoriatic arthritis; RA, rheumatoid arthritis; USpA, undifferentiated spondyloarthropathy.

**Table 4:** Comparison of histological markers between SpA (PsA + AS + USpA) synovium and RA synovium

Marker	SpA (*n *= 50)	RA (*n *= 52)	*P*
Lining layer thickness	1.0 (1.0–2.5)	2.0 (0–3.0)	0.006
Vascularity	2.0 (1.0–3.0)	1.5 (1.0–3.0)	<0.001
Cellular infiltration	1.5 (0–3.0)	2.0 (0–3.0)	0.120
Lymphoid follicles	0 (0–3.0)	0 (0–3.0)	0.232
Plasma cells	0.5 (0–3.0)	0.5 (0–3.0)	0.497
Polymorphonuclear cells	0 (0–3.0)	0 (0–2.5)	0.008
CD3	1.5 (0–3.0)	2.0 (0–3.0)	0.152
CD4	1.0 (0–3.0)	1.0 (0–3.0)	0.192
CD8	1.0 (0–3.0)	1.5 (0–3.0)	0.737
CD20	1.0 (0–3.0)	2.0 (0–3.0)	0.231
CD38	1.5 (0–3.0)	2.0 (0–3.0)	0.060
CD138	1.0 (0–3.0)	2.0 (0–3.0)	0.447
CD68 lining	1.0 (0–3.0)	1.0 (0–3.0)	0.631
CD68 sublining	1.5 (0–3.0)	1.5 (0.5–3.0)	0.957
CD163 lining	2.0 (0–3.0)	1.5 (0–3.0)	0.033
CD163 sublining	1.5 (0–3.0)	1.0 (0–2.5)	0.031
CD1a	0 (0–3.0)	1.0 (0–3.0)	0.121
CD83	0 (0–1.0)	0 (0–3.0)	0.006
CD146	2.0 (0–3.0)	0 (0–3.0)	0.075
α_V_β_3 _lining	1.0 (0–3.0)	1.0 (0–3.0)	0.397
α_V_β_3 _sublining	1.0 (0–3.0)	1.0 (0–3.0)	0.306
E-selectin blood vessels	1.0 (0–3.0)	1.5 (0–3.0)	0.760
ICAM-1 lining	3.0 (1.0–3.0)	2.5 (0–3.0)	0.025
ICAM-1 sublining	2.0 (0–3.0)	2.0 (0–3.0)	0.654
ICAM-1 blood vessels	2.5 (0.5–3.0)	2.5 (0–3.0)	0.652
VCAM-1 lining	3.0 (0–3.0)	3.0 (0–3.0)	0.077
VCAM-1 sublining	1.5 (0–3.0)	1.5 (0–3.0)	0.473
VCAM-1 blood vessels	1.0 (0–3.0)	1.5 (0–3.0)	0.997
Intracellular citrullinated proteins	0%	44%	<0.001
Mab-12A	0%	46%	<0.001

The Mann–Whitney U-test was used to evaluate markers scored semiquantitatively, and the χ^2 ^test was used to evaluate markers scored as present or absent. Results are expressed as median (range), and markers evaluated as present or absent are expressed as percentage of positive results. *P *< 0.05 was considered statistically significant. AS, ankylosing spondylitis; ICAM, intercellular adhesion molecule; PsA, psoriatic arthritis; RA, rheumatoid arthritis; USpA, undifferentiated spondyloarthropathy; VCAM, vascular cell adhesion molecule.

**Table 5:** Comparison of histological markers between oligoarticular and polyarticular PsA

Marker	Oligoarticular PsA (*n *= 28)	Polyarticular PsA (*n *= 17)	*P*
Lining layer thickness	1.0 (1.0–3.0)	1.0 (1.0–2.0)	0.492
Vascularity	1.5 (0–3.0)	2.0 (0–3.0)	0.661
Polymorphonuclear cells	0 (0–3.0)	0 (0–2.5)	0.126
CD68 lining	2.0 (0–3.0)	2.0 (0–2.0)	0.478
CD68 sublining	1.5 (0–3.0)	1.0 (0–3.0)	0.845
CD163 lining	2.0 (0–3.0)	2.0 (0.5–3.0)	0.768
CD163 sublining	1.5 (0–2.5)	1 (0–2.5)	0.935
E-selectin blood vessels	0 (0–3.0)	0.25 (0–2.5)	0.594
Intracellular citrullinated proteins	0%	0%	1.000
Mab-12A	0%	0%	1.000

The Mann–Whitney U-test was used to evaluate markers scored semiquantitatively, and the χ^2 ^test was used to evaluate markers scored as present or absent. Results are expressed as median (range), and markers evaluated as present or absent are expressed as percentage of positive results. *P *< 0.05 was considered statistically significant. PsA, psoriatic arthritis.
